# 2‐Deoxy‐D‐glucose impedes T cell–induced apoptosis of keratinocytes in oral lichen planus

**DOI:** 10.1111/jcmm.16964

**Published:** 2021-10-21

**Authors:** Fang Wang, Jing Zhang, Gang Zhou

**Affiliations:** ^1^ The State Key Laboratory Breeding Base of Basic Science of Stomatology (HubeiMOST) and Key Laboratory of Oral Biomedicine Ministry of Education School and Hospital of Stomatology Wuhan University Wuhan China; ^2^ Department of Oral Medicine School and Hospital of Stomatology Wuhan University Wuhan China

**Keywords:** 2‐deoxy‐D‐glucose, apoptosis, glycolysis, interferon‐γ, keratinocyte, mammalian target of rapamycin, oral lichen planus, T cell

## Abstract

Oral lichen planus (OLP) is a T cell–mediated immunoinflammatory disease. Glycolysis plays an essential role in T‐cell immune responses. Blocking glycolytic pathway in activated T cells represents a therapeutic strategy for restraint of immunologic process in autoimmune disorders. 2‐Deoxy‐D‐glucose (2‐DG) has been widely used to probe into glycolysis in immune cells. This study was aimed to explore the role of glycolysis inhibition by 2‐DG on regulating immune responses of OLP‐derived T cells. We observed that lactic dehydrogenase A (LDHA) expression was elevated in OLP lesions and local T cells. 2‐DG inhibited the expression of LDHA, p‐mTOR, Hif1α and PLD2 in T cells; meanwhile, it decreased proliferation and increased apoptosis of T cells. T cells treated by 2‐DG showed lower LDHA expression and elevated apoptosis, resulting in a reduced apoptotic population of keratinocytes that were co‐cultured with them, which was related to the decreased levels of IFN‐γ in co‐culture system. Rapamycin enhanced the effects of 2‐DG on immune responses between T cells and keratinocytes. Thus, these findings indicated that OLP‐derived T cells might be highly dependent upon high glycolysis for proliferation, and 2‐DG treatment combined with rapamycin might be an option to alleviate T‐cell responses, contributing to reducing apoptosis of keratinocytes.

## 1 INTRODUCTION

Oral lichen planus (OLP) is a chronic inflammatory immune disease of unknown aetiology and has been recognized as an oral potentially malignant disorder (OPMD).^1^, ^2^ The typically histopathological characteristics of OLP are dense subepithelial infiltration of lymphocytes and liquefactive degeneration in the basal keratinocytes.^2^ T cell–mediated dysfunctional immunity has been widely supposed to be the main pathogenesis of OLP.^3^, ^4^ Our previous studies demonstrated that Th1‐biased responses participated in the development of OLP.^5^, ^6^ Specifically, T helper (Th) cells are activated after antigen presentation by major histocompatibility complex II (MHC‐II) molecules and then produce pro‐inflammatory cytokines, including interferon‐γ (IFN‐γ) and interleukin‐2 (IL‐2).^4^ These cytokines together with MHC‐I molecules on keratinocytes activate cytotoxic T cells, which further induce the apoptosis of keratinocytes.^4^

The metabolic reprogramming in T‐cell immune responses is characterized by a switch to aerobic glycolysis, also known as ‘Warburg effect’ that was first described in cancer.^7^ Blocking aerobic glycolysis is pernicious to proliferation and differentiation of effector T cells while beneficial for development of regulatory T cells.^8^ Our recent study showed that activated T cells required glycolytic metabolism to increase glucose utilization, during which Glut1 expression was elevated, thereby promoting T‐cell proliferation and differentiation.^9^ In this process, glucose‐derived pyruvate was guided into glycolytic flux by lactic dehydrogenase A (LDHA), rather than into tricarboxylic acid cycle by pyruvate dehydrogenase (PDH).^9^, ^10^ Moreover, molecular pathways, including mammalian target of rapamycin (mTOR), hypoxia‐inducible factor 1α (HIF1α) and phospholipase D2 (PLD2), motivate glycolytic metabolism in T cells.^11^, ^12^, ^13^ We have found that the expression of p‐mTOR, HIF1α and PLD2 was elevated in peripheral or local T cells of OLP, and mTOR/HIF1α/PLD2 axis could upregulate the phosphorylation of LDHA in T cells.^9^, ^14^, ^15^ Besides, mTOR pathway could dynamically respond to extracellular glucose signals, then correspondingly regulate T‐cell glycolysis, and finally support T‐cell proliferation and differentiation.^9^ These findings suggested that deregulated mTOR pathway might disturb the glycolytic metabolism in T cells, thereby leading to the dysfunctional immunity of OLP. Lactic dehydrogenase activity has been investigated in OLP lesions in 1965^16^; however, LDHA expression and the role of glycolysis in T cells from OLP remains unknown to date.

Recently, immunometabolism has been a burgeoning field.^8^, ^17^, ^18^ Targeting aerobic glycolysis turns into an attractive therapeutic strategy for restraint of immunologic process in autoimmune diseases, such as systemic lupus erythematosus (SLE) and rheumatoid arthritis (RA).^7^, ^10^ This therapy does not have broad toxicity in normal tissues that depend on oxidative metabolism.^7^ 2‐Deoxy‐D‐glucose (2‐DG) is a glucose analogue that blocks the initial phase of glycolysis. Fu et al. demonstrated that 2‐DG significantly reduced CD4^+^ T‐cell infiltration and attenuated Sjögren's syndrome (SS)‐like autoimmune response in mice model.^19^ Thus, targeting aerobic glycolysis by 2‐DG is a feasible strategy for controlling T‐cell responses in diseases.

In this study, the expression of LDHA in OLP lesions and local T cells was investigated by immunohistochemistry and immunofluorescence staining. Then, the effects of 2‐DG on OLP‐derived T cells were assessed by detecting the expression of LDHA and mTOR pathway, T‐cell proliferation, and cell apoptosis. Finally, the co‐culture system was established with 2‐DG pretreated OLP‐derived T cells and LPS‐stimulated keratinocytes to explore the modulation of 2‐DG on immune responses between T cell and keratinocytes.

## 2 MATERIALS AND METHODS

### 2.1 Patients

Sixteen patients with diagnosis of OLP and ten healthy controls were enrolled prospectively in this study. They were referred to the Department of Oral Medicine, School and Hospital of Stomatology, Wuhan University. Diagnosis of OLP was made according to the clinical and histopathological findings.^2^ Healthy controls came from patients without any impairment on oral mucosa or systemic diseases. Other inclusion and exclusion standards were consistent to our previous study.^9^ The clinical characteristics of samples were shown in Appendix S1 and Appendix S2. This research was carried out on the principles of the Declaration of Helsinki. It has been approved by the Ethical Committee Broad of School and Hospital of Stomatology, Wuhan University (no. 2015C35). The patients’ informed consent was obtained before collection of tissue specimens and peripheral blood.

### 2.2 Immunohistochemistry and immunofluorescence

The formalin‐fixed, paraffin‐embedded oral mucosal tissues were prepared, sectioned at 4 micrometres and analysed for the presence of LDHA antigen on the basis of standard immunohistochemistry protocols. Tissue sections were incubated overnight at 4℃ with LDHA rabbit polyclonal antibody (1:100, Catalog # 19987–1‐AP, Proteintech, Wuhan, China), followed by HRP polymer conjugated anti‐rabbit secondary antibody for 1 h at 37°C. Next, diaminobenzidine solution was used to detect the signal, and haematoxylin was used to counterstain the nucleus. Images were acquired using OLYMPUSCH30 microscope (BHS‐313, Olympus, Japan) at 200× and 400×. The mean optical density (MOD) was used to analyse the positive staining via Image‐Pro Plus 6.0 version (IPP, Media Cybernetics).

The sections for immunofluorescence staining were incubated with CD3 mouse monoclonal antibody (1: 100, Catalog # 60181–1‐Ig, Proteintech) and LDHA rabbit polyclonal antibody (1: 100, Proteintech) at 4°C overnight. Then, signals were observed using Cy3‐conjugated (1: 200, Catalog # GB21301, Servicebio, Wuhan, China) and Alexa Fluor® 488‐conjugated (1: 200, Catalog # GB25303, Servicebio) secondary antibodies. Nuclei were stained with DAPI. The slides were scanned under Pannoramic MIDI, and images were captured with CaseViewer (3DHISTECH). The fluorescence contrast parameters were described in detail in Appendix S3.

### 2.3 Cell culture

Peripheral blood mononuclear cells (PBMCs) were isolated from four erosive OLP patients’ blood samples using Ficoll‐Hypaque solution (Catalog # LDS1075, TBD) by density gradient centrifugation. Next, T cells were sorted from PBMCs using BD IMag™ Human T Lymphocyte Enrichment Set‐DM (Catalog # 557874) by IMag™ cell separation system (BD). T cells (1 × 10^6^ cells/ml) were cultured with human T‐cell robust expansion medium (Catalog # CT‐007, StemEry) and 500 U/ml IL‐2 (Catalog # I7908, Sigma). The medium was replaced by half every 2 days. Subsequently, T cells (4.5 × 10^5^ cells/ml) were treated with 1 μg/ml anti‐CD3 mAb and 2 μg/ml anti‐CD28 mAb (Catalog # 16–0037–85, # 16–0289–85, Thermo, Massachusetts, USA) for 72 h in a 37°C humidified incubator with 5% CO_2_. Then, activated T cells (1 × 10^6^ cells/ml) were cultured with 2‐DG (1 or 5 mM, Catalog # B1027, APExBIO)) or/and rapamycin (100 nM, Catalog # A8167, APExBIO) for 24 h. 2‐DG and rapamycin were dissolved in dimethylsulfoxide (DMSO).

Human oral keratinocytes (cell line named OKF4, 5 × 10^4^ cells/ml) were seeded in a 24‐well plate with keratinocyte serum‐free medium (K‐SFM, Catalog # 10744019, Thermo) at 37°C with a 5% CO_2_. In the logarithmic growth phase, keratinocytes were stimulated with 10 μg/ml LPS (Catalog # L2654, Sigma) for 36 h to simulate a local inflammatory microenvironment of OLP and resuspended with fresh medium before co‐culture.

The co‐culture system was established with OLP‐derived T cells and human oral keratinocytes via 24‐well transwell inserts with aperture of 0.4 μm (Catalog # 140620, Thermo). OLP‐derived T cells (1 × 10^6^ cells/ml) were pretreated with 5 mM 2‐DG or/and 100 nM rapamycin for 24 h, then placed in the upper compartment with fresh medium and co‐cultured with LPS‐stimulated keratinocytes from the lower compartment for 24 h. The co‐culture medium consisted of 500 μl human T‐cell robust expansion kit and 500 μl K‐SFM.

### 2.4 Immunocytochemistry

Paraformaldehyde‐fixed cells were smeared on the slides, permeabilized with Triton X‐100 after dying at room temperature, blocked with bull serum albumin (3%), and incubated with a primary antibody against LDHA (Proteintech) and then Cy3‐conjugated secondary antibody (Catalog # GB21303, Servicebio). Nuclei were stained with DAPI. The cell smears were observed under a fluorescence microscope (NIKON ECLIPSE C1, Japan) and an imaging system (NIKON DS‐U3). The images were captured with CaseViewer (3DHISTECH).

### 2.5 Western blotting

Protein extraction was performed with RIPA buffer (Catalog # P0013B, Beyotime) by centrifugation at 12,000 × *g* for 20 min. Proteins were separated by 8%, 10% or 12% polyacrylamide gel electrophoresis and transferred on polyvinylidene fluoride membranes. After blocking with 5% skimmed milk, membranes were incubated overnight at 4°C in the presence of primary antibodies against mTOR (1:1000, Catalog # 2983S, CST), p‐mTOR (1:1000, Catalog # 5536S, CST), 4E‐BP1 (1:1000, Catalog # 9644S, CST), p‐4E‐BP1 (1:1000, Catalog # 2855S, CST), HIF1α (1:1000, Catalog # 20960–1‐AP, Proteintech), PLD2 (1:1000, Catalog # NBP2‐61784, Novus) and β‐actin (1:1000, Catalog # BM0627, Bosterbio). Horseradish peroxidase‐conjugated secondary antibodies were subsequently incubated, and the signals were visualized with enhanced chemiluminescence by the Odyssey^®^ Fc Imaging System (LI‐COR). The grey‐scale analysis was conducted via ImageJ software (National Institutes of Health, Bethesda, Maryland, USA).

### 2.6 Cell proliferation assay

Primary T cells at a density of 5 × 10^4^ cells/well were seeded into 96‐well plates for 24 h. After treatment, the cells were incubated with the Cell Counting Kit‐8 (CCK‐8, Catalog # CK04, Dojindo) reagent for 2.5 h at 37°C. Cell viability was quantified by optical density (OD) value at an absorbance wavelength of 450 nm.

### 2.7 Cell apoptosis assay

Either T cells or keratinocytes were resuspended at a density of 10^6^ cells/ml approximately for the apoptosis assay and assessed using an Annexin V‐FITC/PI Apoptosis Detection Kit (Catalog # KGA108, KeyGEN) by flow cytometry (Beckman). The results were expressed as the percentage of apoptotic (Annexin V positive) cells.

### 2.8 Cytokines assay

The concentrations of IFN‐γ, IL‐4 and IL‐17 in the supernatants of co‐culture system were measured using human IFN‐γ, IL‐4 and IL‐17 ELISA kits (Catalog # CHE0017, # CHE0005, # CHE0054, 4A Biotech Co., Ltd).

### 2.9 Statistical analysis

Statistical analyses were performed, and graphs were generated using the Graphpad Prism 5 (Graphpad Software). The data were reported as the mean ± SEM. Significant differences between groups were evaluated by Mann‐Whitney test, unpaired *t* test and one‐way ANOVA. *p *< 0.05 was regarded as statistical significance level.

## 3 RESULTS

### 3.1 The glycolysis was highly activated in OLP lesions and local T cells

**FIGURE 1:**
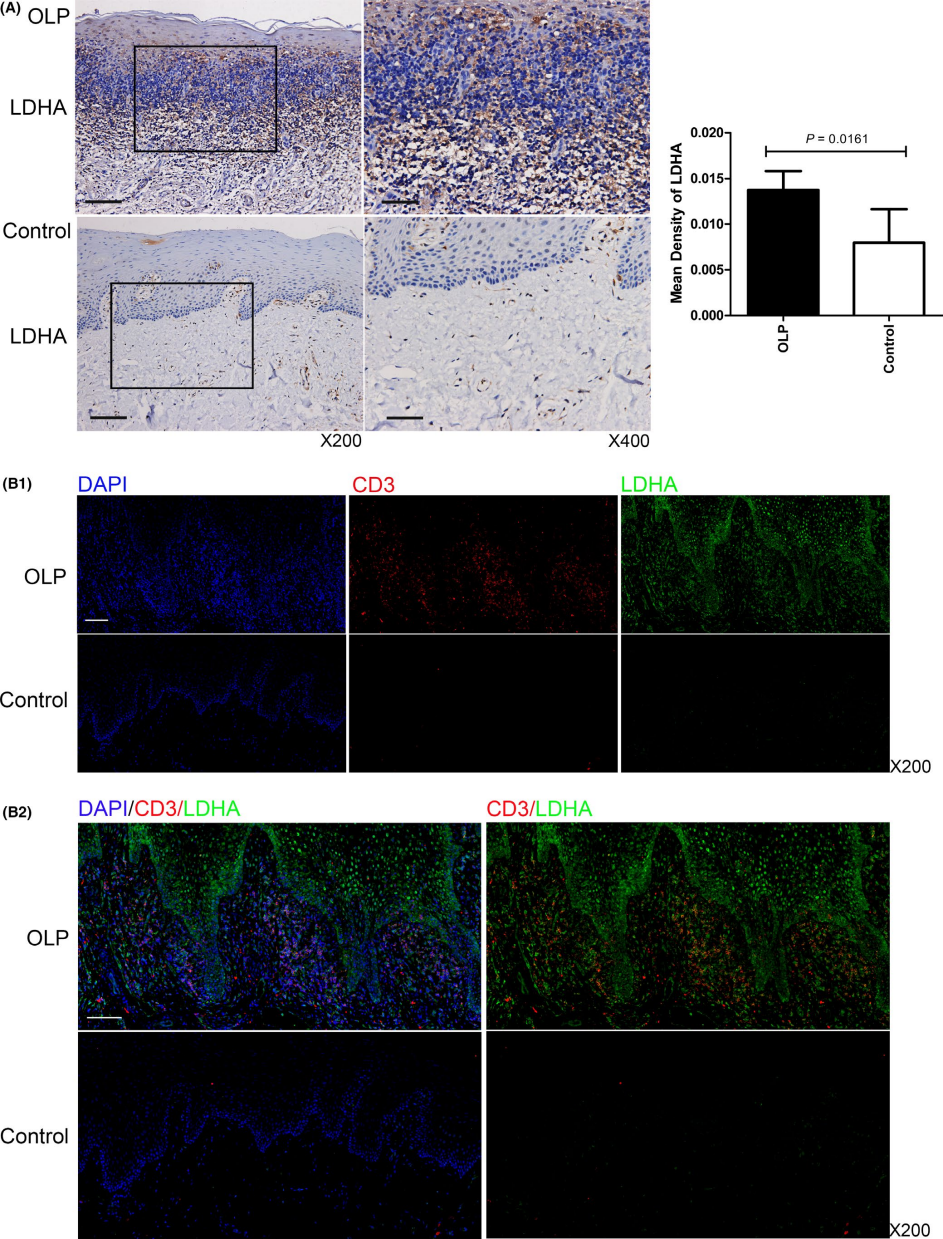
LDHA protein was highly expressed in OLP lesions and local T cells. Representative images of immunohistochemical staining and immunofluorescent staining for LDHA in OLP lesions (*n* = 12) and normal oral mucosa tissues (n = 10). (A) LDHA immunoreactivity was observed in the epithelium and infiltrated lymphocytes of OLP lesions. The statistical data were reported using mean optical density (MOD) of at least three randomly captured fields at ×400 magnification. Bar: 100 μm for ×200, 50 μm for ×400. (B) Abundant red fluorescence signals for CD3 overlapped with the green signals for LDHA in OLP lesions. b1: single channel images for DAPI (blue), CD3 (red), LDHA (green); b2: merged images. Bar: 100 μm for ×200

LDHA can guide glucose metabolism into the glycolysis pathway.^10^ Therefore, the glycolysis in OLP lesion and local T cells was evaluated by detecting LDHA protein. The diaminobenzidine staining and immunostaining data showed that LDHA resided in the cytoplasm and nuclei of cells (Figure 1). In Figure 1a, the diaminobenzidine staining was observed in the epithelium of OLP lesions and predominantly accentuated in the basal layers, where lymphocytes were infiltrated. For healthy tissues, there was slight staining in the epithelium and inflammatory cells in connective tissue. The statistical data showed that OLP lesions had significantly increased MOD values of LDHA (*p* = 0.0161). Besides, MOD value of LDHA staining was elevated in erosive OLP lesions, compared to non‐erosive OLP lesions and healthy tissues (Appendix S4). Immunofluorescent staining showed abundant red signals for CD3 overlapped with the green signals for LDHA in OLP lesions, confirming that CD3^+^ T cells in OLP lesions expressed a large number of LDHA protein (Figure 1b).

### 3.2 OLP‐derived T cells were dependent upon high glycolysis and mTOR pathway for proliferation

**FIGURE 2:**
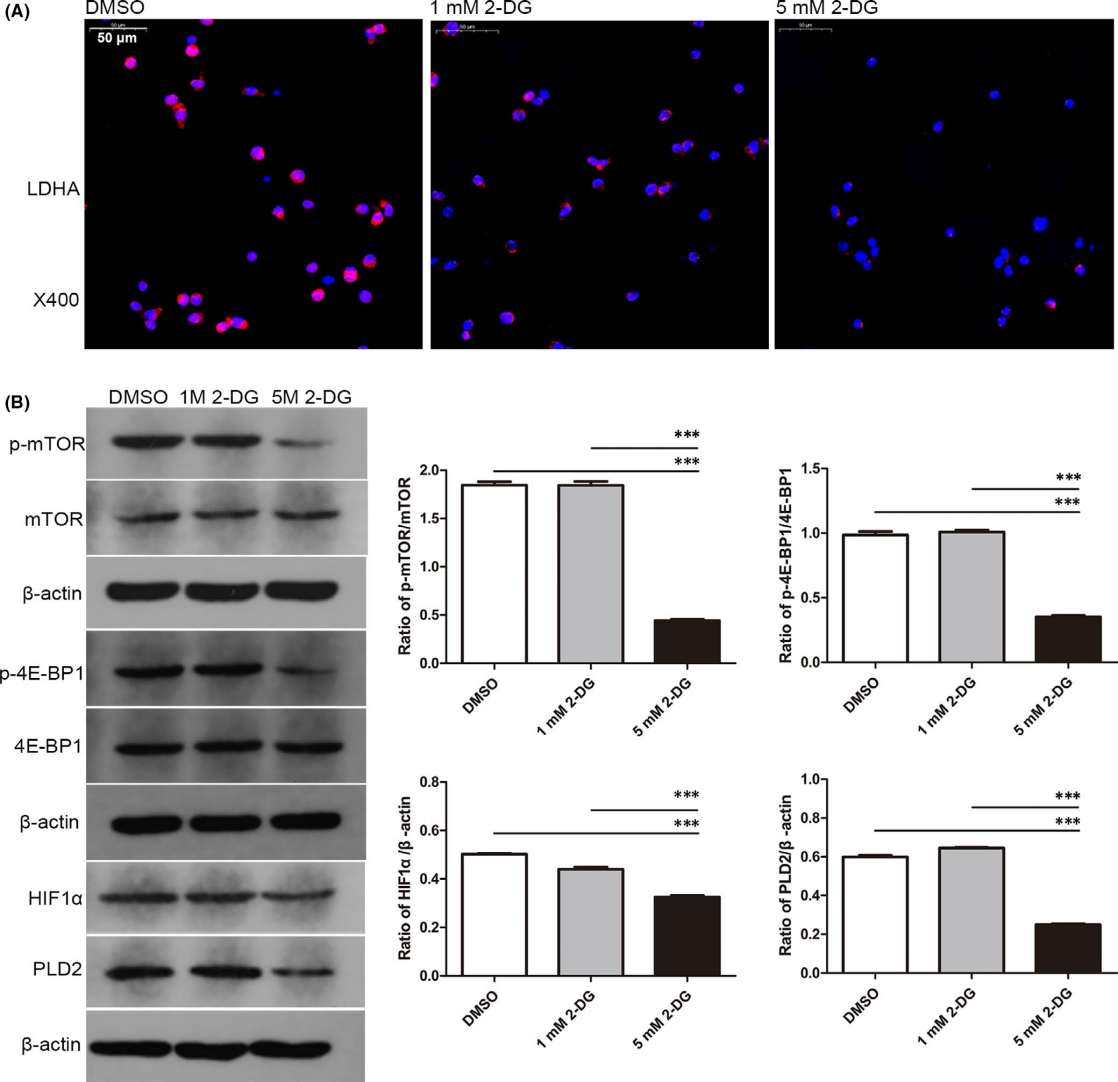
2‐DG inhibited glycolysis and mTOR pathway in OLP‐derived T cells. OLP‐derived T cells were activated with 1 μg/ml anti‐CD3 mAb and 2 μg/ml anti‐CD28 mAb for 72 h, then cultured with 1 mM or 5 mM 2‐DG for 24 h. 2‐DG was dissolved in DMSO. DMSO at a concentration of 0.1% (v/v) was used as the solvent control. (A) Representative images of immunofluorescent staining for LDHA expression in T cells. The fluorescence intensity of LDHA was decreased in the 2‐DG groups. 5 mM 2‐DG group showed a weaker fluorescence than 1 mM 2‐DG group. Red: LDHA. Blue: nucleus. Bar: 50 μm for ×400. (B) Representative images of Western Blot detection for p‐mTOR, mTOR, p‐4E‐BP1, 4E‐BP1, HIF1α and PLD2 expression. Grey‐scale analysis revealed that the ratios of p‐mTOR/mTOR, p‐4E‐BP1/4E‐BP1, HIF1α/β‐actin and PLD2/β‐actin were decreased in 5 mM 2‐DG group, rather than in 1 mM 2‐DG group. ***, *p* < 0.001

**FIGURE 3:**
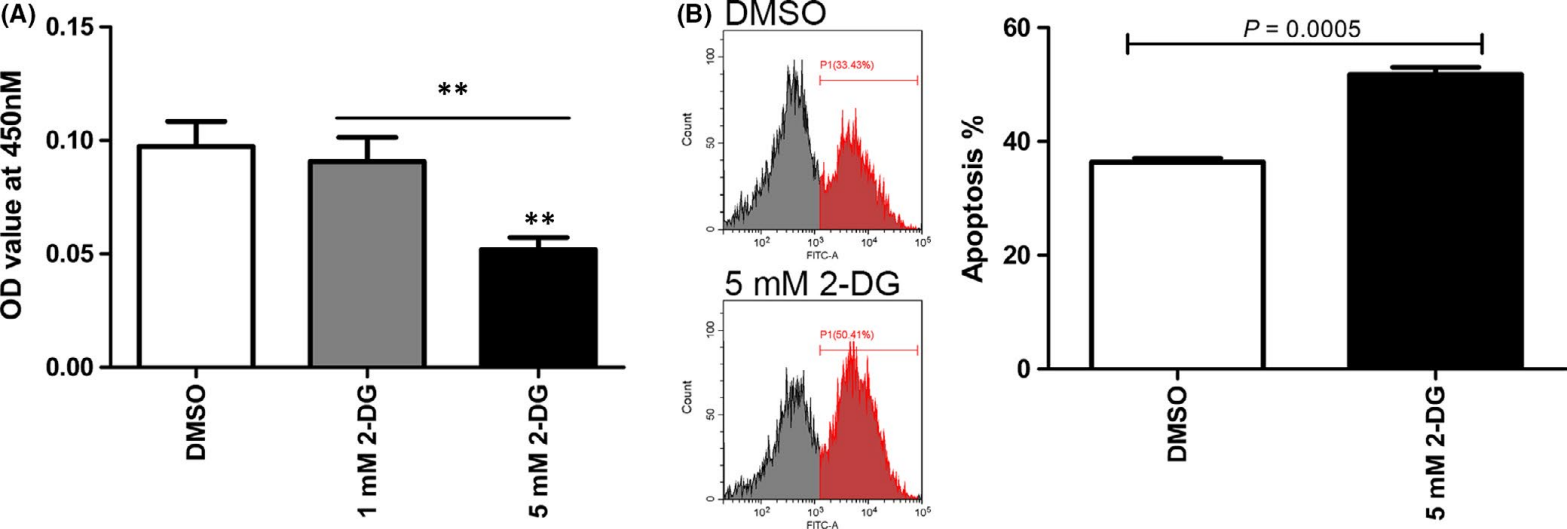
2‐DG decreased proliferation and increased apoptosis of OLP‐derived T cells. (A) After activation, OLP‐derived T cells were cultured with 1 or 5 mM 2‐DG for 24 h for proliferation analysis using CCK‐8 reagents. The OD value in 5 mM 2‐DG group was lower than that in 1 mM 2‐DG group and DMSO group. **, *p* < 0.01. (B) OLP‐derived T cells were cultured with 5 mM 2‐DG for 24 h for apoptosis analysis using Annexin V‐FITC/PI reagents, and representative images for T‐cell apoptosis were shown. The number of Annexin V‐positive cells in 5 mM 2‐DG group was larger than that in DMSO group. DMSO at a concentration of 0.1% (v/v) was used as the solvent control group

2‐DG, a glycolytic inhibitor, was used to study the effects of glycolysis on OLP‐derived T cells. At concentrations of 1 and 5 mM, 2‐DG treatment for 24 h visibly decreased the fluorescence intensity of LDHA in T cells (Figure 2a). 5 mM 2‐DG group showed a weaker fluorescence than 1 mM 2‐DG group. These findings indicated that 1 and 5 mM 2‐DG could effectively inhibit glycolysis of OLP‐derived T cells. For mTOR pathway, 5 mM 2‐DG remarkably downregulated the ratios of p‐mTOR/mTOR, p‐4E‐BP1/4E‐BP1, HIF1α/β‐actin and PLD2/β‐actin in OLP‐derived T cells, while 1 mM 2‐DG did not (*p* < 0.001, Figure 2b). Meantime, 5 mM 2‐DG significantly inhibited the proliferative activity of OLP‐derived T cells, rather than 1 mM 2‐DG (*p* < 0.01, Figure 3a). Taken together, when both glycolysis and mTOR pathway were inhibited, 2‐DG could impede the proliferation of OLP‐derived T cells. Further apoptosis analysis revealed that the number of apoptotic OLP‐derived T cells in 5 mM 2‐DG group was larger than that in DMSO group (*p* = 0.0005, Figure 3b).

### 3.3 2‐DG treatment combined with rapamycin alleviated T‐cell responses, contributing to the reduced apoptosis of keratinocytes in co‐culture system

**FIGURE 4:**
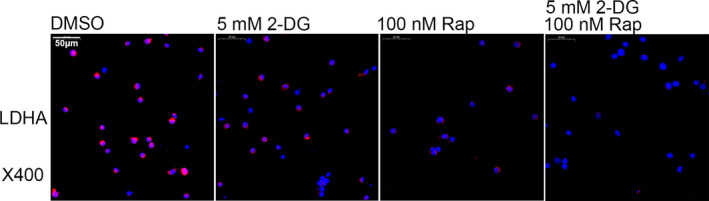
2‐DG and/or rapamycin inhibited LDHA expression in OLP‐derived T cells. Representative images of immunofluorescent staining for LDHA expression. DMSO at a concentration of 0.1% (v/v) was used as the solvent control group. Red: LDHA. Blue: nucleus. Bar: 50 μm for ×400

**FIGURE 5:**
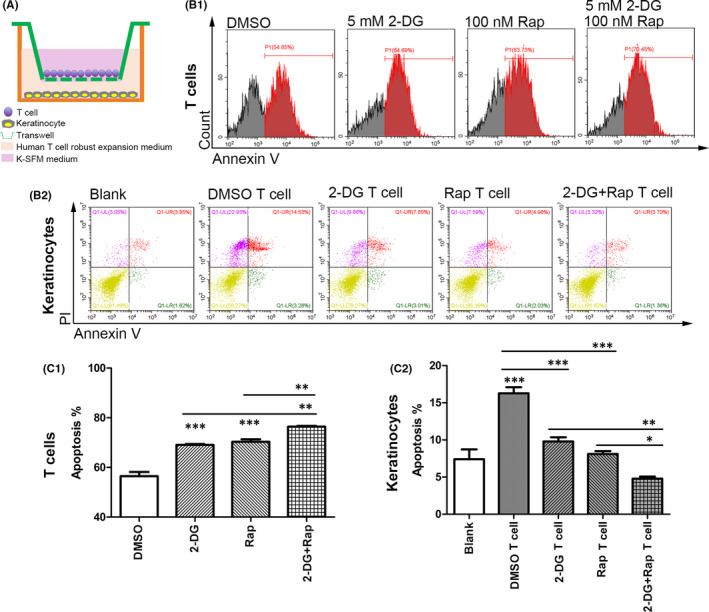
Blocking glycolysis and mTOR pathway in OLP‐derived T cells resulted in a reduced apoptotic population of keratinocytes that were co‐cultured with them. (A) The co‐culture system was established with OLP‐derived T cells and human oral keratinocytes via transwell inserts. T cells were pretreated with DMSO (at 0.1% v/v), 5 mM 2‐DG or 100 nM rapamycin for 24 h. Then, T cells were placed in the upper compartment with fresh medium; LPS‐stimulated keratinocytes were cultured in the lower compartment. They were co‐cultured for 24 h for apoptosis analysis. The co‐culture medium was consisted of 500 μl human T‐cell robust expansion kit and 500 μl K‐SFM. For T cells, DMSO at a concentration of 0.1% (v/v) was used as the solvent control group. For keratinocytes, cells cultured without T cells were used as a blank control. (B) Flow cytometry assay was used to detect the apoptosis of OLP‐derived T cells and keratinocytes in co‐culture system. B1: representative images for T‐cell apoptosis; B2: representative images for keratinocyte apoptosis. (C) The histogram of apoptosis rates of T cells and keratinocytes in co‐culture system. C1: for T cells, the apoptotic populations in 2‐DG group and Rap group were larger than DMSO group, and the apoptotic populations in 2‐DG +Rap group were larger than 2‐DG group and Rap group. C2: for keratinocytes, the apoptosis rate was increased in DMSO T‐cell group compared to Blank group, while decreased in 2‐DG T‐cell group and Rap T‐cell group compared to DMSO T‐cell group. The apoptosis rate in 2‐DG +Rap T‐cell group was lower than that in 2‐DG T‐cell group and Rap T‐cell group. *p < 0.05; **p < 0.01; ***p < 0.001

In OLP lesions, colloid bodies were seen in basement membrane of OLP tissues rather than in healthy controls (Appendix S5). Colloid bodies represented apoptotic keratinocytes, suggesting that apoptosis is common in the basal layer of OLP. The mTOR inhibitor rapamycin was used in combination with 2‐DG to investigate the effects of glycolysis and mTOR pathway on T cell–mediated apoptosis of keratinocytes. 2‐DG and rapamycin both inhibited the expression of LDHA in OLP‐derived T cells, and the combined treatment showed the strongest inhibitory effects (Figure 4). In co‐culture system (Figure 5a), keratinocytes that were cultured with OLP‐derived T cells had elevated apoptotic populations compared to ones cultured alone (Figure 5b2 & c2, DMSO T‐cell group vs. Blank group, *p* < 0.001). 2‐DG or rapamycin pretreatment increased the apoptotic populations in OLP‐derived T cells (Figure 5b1 & c1, 2‐DG group vs. DMSO group, *p* < 0.001; Rap group vs. DMSO group, *p* < 0.001), correspondingly the apoptosis rates of keratinocytes were decreased when co‐cultured with T cells that were pretreated with 2‐DG or rapamycin (Figure 5b2 & c2, 2‐DG T‐cell group vs. DMSO T‐cell group, *p* < 0.001; Rap T‐cell group vs. DMSO T‐cell group, *p* < 0.001). Combined treatment of 2‐DG and rapamycin resulted in a higher apoptosis rate of OLP‐derived T cells and lower apoptotic events of keratinocytes after co‐culture (Figure 5b & c). Specifically, 2‐DG and/or rapamycin mainly increased the early apoptosis of T cells, while T cells predominantly induced the late apoptosis of keratinocytes (Appendix S6).

### 3.4 IFN‐γ level in co‐culture system was decreased after inhibiting glycolysis and mTOR pathway in OLP‐derived T cells

**FIGURE 6:**
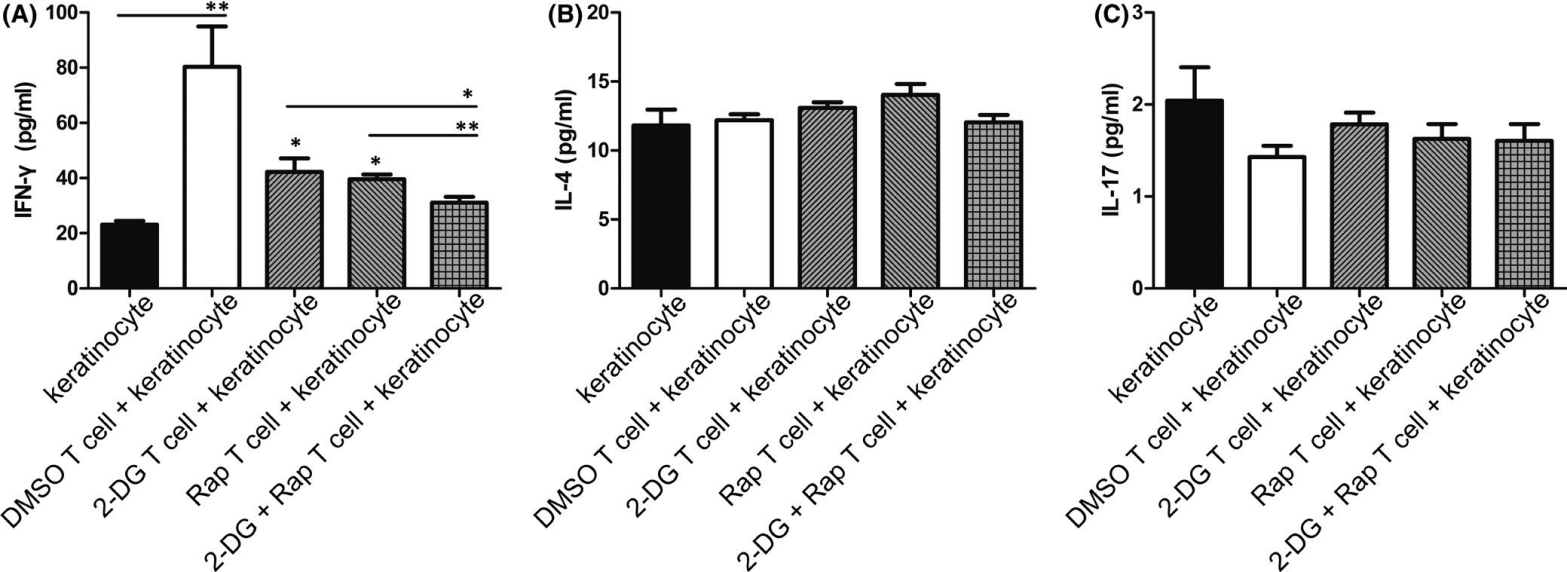
IFN‐γ level in co‐culture system was decreased after blocking glycolysis and mTOR pathway in OLP‐derived T cells. (A, B, C) ELISA kits were used to detect the levels of IFN‐γ, IL‐4 and IL‐17 in co‐culture system. IFN‐γ level was increased in DMSO T‐cell +keratinocyte group compared to keratinocyte group, while decreased in 2‐DG T‐cell +keratinocyte group and Rap T‐cell +keratinocyte group compared to DMSO T‐cell +keratinocyte group. IFN‐γ level in 2‐DG +Rap T‐cell +keratinocyte group was lower than that in 2‐DG T‐cell +keratinocyte group and Rap T‐cell +keratinocyte group. *p < 0.05; **p < 0.01

Cytokines play an important role in T cell–mediated keratinocyte apoptosis.^4^ Considering the involvement of Th1, Th2 and Th17 cells in the immunopathogenesis of OLP, the levels of IFN‐γ, IL‐4 and IL‐17 in medium of co‐culture system were detected. Results showed that IFN‐γ level was increased when keratinocytes were co‐cultured with OLP‐derived T cells (Figure 6a), which was corresponded to the results of apoptotic analysis for T cells and keratinocytes in Figure 5. When OLP‐derived T cells were pretreated with 2‐DG or rapamycin, the IFN‐γ levels in co‐culture system were decreased, and combined treatment showed the strongest effects on the IFN‐γ level. No significant change was found in the levels of IL‐4 and IL‐17 in the co‐culture supernatant (Figure 6b,c).

## 4 DISCUSSION

The abnormal metabolic state of T cells interferes in cellular biology and functions, thereby results in dysfunctional immune responses, ultimately leads to chronic inflammation in autoimmune diseases, including systemic lupus erythematosus (SLE) and rheumatoid arthritis (RA).^10^ It has been reported that CD4^+^ T cells in SLE have increasing abnormal mitochondria which cause hyperactivated metabolism, and naïve T cells in RA undertake the pentose phosphate pathway and possess anti‐glycolysis effects.^20^, ^21^, ^22^ In different T cell–mediated diseases, metabolic status is different. However, little is known about metabolic state of T cells in OLP. In this study, data showed that expression of LDHA was elevated in OLP lesions and local T cells compared with healthy tissues, which was consistent the results reported by Shklar that lactic dehydrogenase activity was elevated in OLP lesions.^16^ LDHA is a glycolytic enzyme that converts glucose‐derived pyruvate into lactic acid.^10^ With lactate production, NAD^+^ pools would backfill and then maintain glycolytic flux.^23^ Therefore, it was demonstrated that glycolysis mediated by LDHA was hyperactivated in T cells of OLP.

2‐DG is considered as an inhibitor of glucose metabolism. Concretely, 2‐DG can be phosphorylated by hexokinase into 2‐deoxyglucose‐6‐phosphate, which cannot continue to be metabolized in the energy payoff phase of glycolysis.^24^ Competitive nature of 2‐DG over glucose utilization and inaccessibility of phosphorylated product into glycolysis can reduce substrates for the glycolytic flux, thus decreasing the production of ATP.^24^, ^25^, ^26^ Intracellular ATP can active mTOR pathway in a variety of ways.^27^ Reyes et al. found that 2‐DG induced the depletion of cellular ATP, contributing to the inhibition of mTOR as well as its downstream 4E‐BP1.^25^ Our previous findings revealed that the activation of mTOR pathway in T cells varied with glucose concentration, demonstrating mTOR serves as an important metabolic sensor.^9^ Besides, inhibition of mTOR pathway could reduce the expression of Hif1α in T cells,^14^ which is a major transcriptional regulator of LDHA. Either downregulation of mTOR or Hif1α could lead to the inhibition of LDHA.^28^, ^29^ In this study, 2‐DG at concentration of 1 and 5 mM both remarkably inhibited the expression of LDHA in OLP‐derived T cells, while only 5 mM 2‐DG significantly reduced activation of mTOR and 4E‐BP1 and expression of Hif1α and PLD2 in OLP‐derived T cells. Meanwhile, the reduced proliferation was observed in OLP‐derived T cells treated with 5 mM 2‐DG, rather than 1 mM 2‐DG. These findings suggested that 2‐DG could impede the proliferation of OLP‐derived T cells when both glycolysis and mTOR pathway were inhibited, that is, mTOR pathway might be responsible for the regulation of 2‐DG on T‐cell proliferation. In our previous studies, it has been reported that the phosphorylation of mTOR was higher in T cells of OLP patients than that in healthy samples.^9^, ^15^ Thus, OLP‐derived T cells required a high concentration of 2‐DG to efficiently block the mTOR pathway and simultaneously enhance the inhibition of glycolysis, thereby impeding T‐cell proliferation and functions.

IFN‐γ is an important cytokine from Th1 cells. We found that the levels of IFN‐γ in serum and local tissues of OLP were higher than healthy controls,^6^ and the transcriptional expression of T‐bet was increased in the peripheral and infiltrating T cells of OLP.^5^, ^30^ The induction of T‐bet is dependent on IFN‐γ signalling and critical for Th1 differentiation, contributing to the immunologic dissonance in OLP.^31^ Moreover, IFN‐γ increases CD40 expression on keratinocytes and promotes these cells releasing chemokines, such as CXCL9, CXCL10 and CXCL11, which may effectively mediate T cells recruitment.^32^ CD40‐expressed cells in OLP lesions were reported to be relatively prone to apoptosis.^33^ Marshall et al. demonstrated that CD154, the CD40 ligand, was expressed on T cells in OLP lesions, and positive cells mainly located near focal areas of epithelial cell damage.^33^ Thus, under the manipulation of IFN‐γ, peripheral T cells would migrate to OLP lesions, bind to keratinocytes, and mediate keratinocyte apoptosis through ligation between CD40 and CD154. On the contrary, IFN‐γ directly induces morphological changes in epithelial cells from polygonal shape to fibroblast‐like shape, loss of cell‐to‐cell contact, E‐cadherin overexpression and downregulated Vimentin expression, which finally results in the liquefactive degeneration of basal keratinocytes in OLP.^34^ Additionally, IFN‐γ‐inducible CCL27 secretion from keratinocytes is involving in the recruitment of memory T cells into epithelium, which might be associated with the recurrence and chronicity of OLP.^35^ In the present study, keratinocytes that were co‐cultured with OLP‐derived T cells had a larger number of apoptotic populations than ones cultured alone. Besides, IFN‐γ was highly expressed in co‐culture supernatant of keratinocytes and OLP‐derived T cells. Collectively, it was postulated that IFN‐γ was involved in the interaction between OLP‐derived T cells and keratinocytes.

**FIGURE 7:**
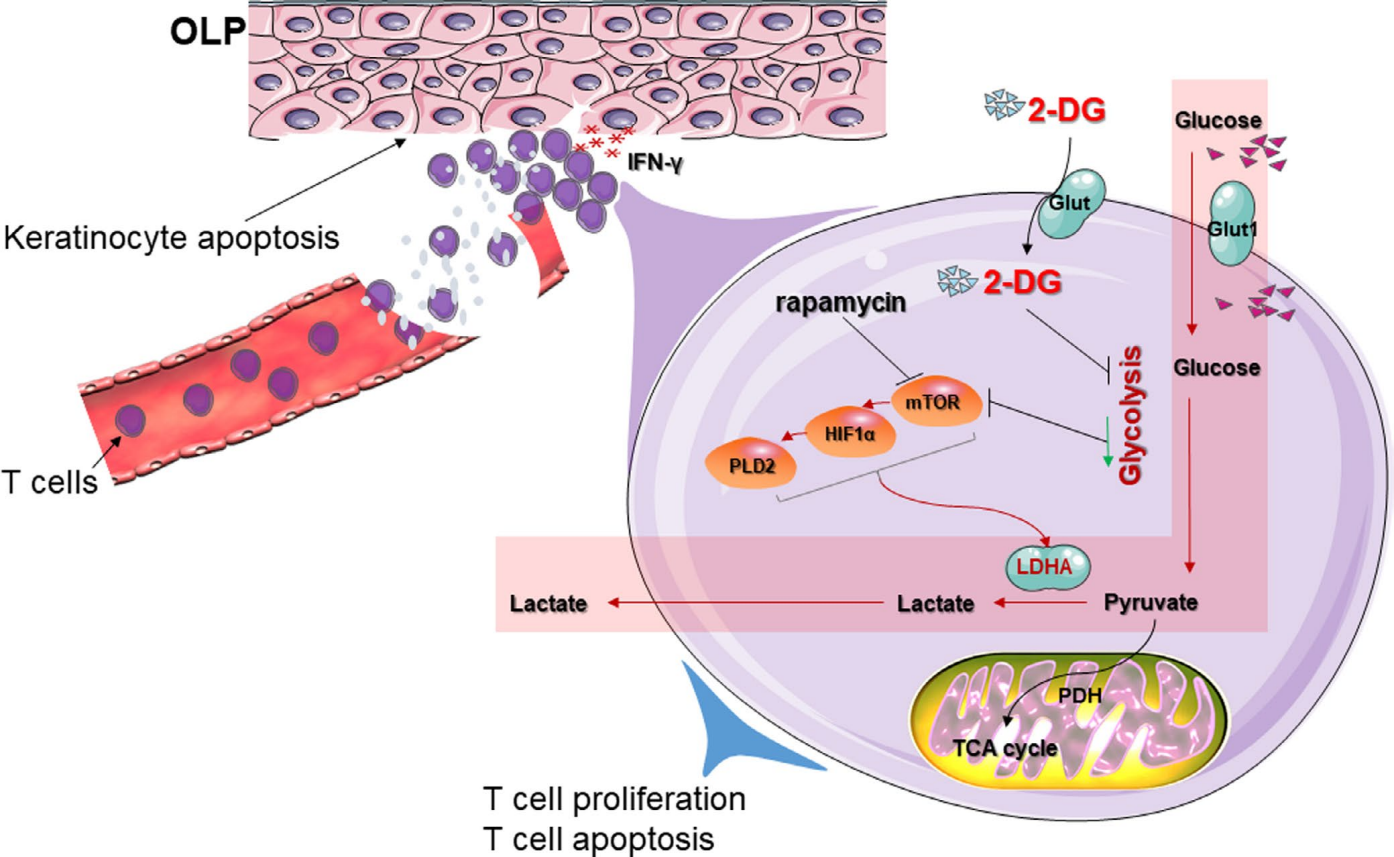
Diagram of the role of 2‐DG on OLP‐derived T cells. OLP T cells are highly dependent upon high glycolysis for proliferation. 2‐DG blocking glycolysis leads to the inhibition of mTOR pathway, which is responsible for the expression of LDHA. T cells treated by 2‐DG have lower LDHA expression and elevated apoptosis, resulting in a reduced apoptotic population of target cells, namely keratinocytes, which is related to the decreased levels of IFN‐γ in the microenvironment. Rapamycin enhanced the effects of 2‐DG on immune responses between T cells and keratinocytes

Glycolysis has been shown to be indispensable for Th1 differentiation and signature cytokine IFN‐γ production.^36^ It was shown that CD4^+^ T cells in the absence of LDHA produced less IFN‐γ.^37^ LDHA promoted IFN‐γ expression through an epigenetic mechanism of histone acetylation, indicating a critical role for LDHA‐mediated glycolysis in promoting Th1 responses.^37^ Moreover, our previous study demonstrated that blocking mTOR pathway could diminish LDHA phosphorylation and reduce T‐bet expression in T cells, suggesting that mTOR‐dependent glycolytic pathway in T cells might serve as a metabolic target to regulate Th1 responses.^9^ The current data showed that IFN‐γ level was increased when keratinocytes were co‐cultured with OLP‐derived T cells. When OLP‐derived T cells were pretreated with 2‐DG or rapamycin, the IFN‐γ levels in co‐culture system were decreased, and combined treatment showed the strongest effects on the IFN‐γ level. These findings suggested that both blocking glycolysis and mTOR pathway in OLP‐derived T cells could effectively reduce IFN‐γ production then weaken the induction for keratinocyte apoptosis (Figure 7).

In conclusion, OLP‐derived T cells might be highly dependent upon high glycolysis for proliferation. 2‐DG treatment combined with rapamycin might be an option to alleviate T‐cell responses, contributing to the reduced apoptosis of keratinocytes, which was related to the decreased levels of IFN‐γ in co‐culture system.

## CONFLICTS OF INTEREST

The authors declared no conflict of interest.

## AUTHOR CONTRIBUTION

**Fang Wang:** Conceptualization (lead); Data curation (lead); Formal analysis (lead); Investigation (lead); Resources (lead); Validation (lead); Writing‐original draft (lead). **Jing Zhang:** Conceptualization (supporting); Funding acquisition (lead); Resources (supporting); Writing‐review & editing (supporting). **Gang Zhou:** Data curation (supporting); Funding acquisition (lead); Resources (supporting); Supervision (supporting); Writing‐review & editing (supporting).

## Supporting information

Appendix S1

Appendix S2

Appendix S3

Appendix S4

Appendix S5

Appendix S6

## Data Availability

Data are available from the corresponding author upon reasonable request.
