# Supplementary material for: 2‐Deoxy‐D‐glucose impedes T cell–induced apoptosis of keratinocytes in oral lichen planus
Source: J Cell Mol Med. 2021 Oct 21;25(21):10257–67. doi: 10.1111/jcmm.16964 (PMC8572795; doi:10.1111/jcmm.16964)
Supplement: Supplementary file 1 — Appendix S1 [file JCMM-25-10257-s001.docx]

| **Appendix 1.** The clinical characteristics of OLP patients and healthy controls | | |
| --- | --- | --- |
|  | **OLP** | **Healthy controls** |
| **Total number** | 16 | 10 |
| **Gender** |  |  |
| Male | 8 | 4 |
| Female | 8 | 6 |
| **Age** |  |  |
| Range | 23 - 64 | 21 - 56 |
| Mean ± SD | 45.0 ± 10.8 | 40.2 ± 11.2 |
| **Medication use** | None | None |
