# Supplementary material for: 2‐Deoxy‐D‐glucose impedes T cell–induced apoptosis of keratinocytes in oral lichen planus
Source: J Cell Mol Med. 2021 Oct 21;25(21):10257–67. doi: 10.1111/jcmm.16964 (PMC8572795; doi:10.1111/jcmm.16964)
Supplement: Supplementary file 2 — Appendix S2 [file JCMM-25-10257-s006.docx]

| **Appendix 2.** The clinical data of OLP patients | | | | |
| --- | --- | --- | --- | --- |
| **Case No.** | **Clinical subtype** | **Gender** | **Age (years)** | **Pathological sites** |
| 1 | erosive OLP | Male | 52 | Dorsum of the tongue |
| 2 | erosive OLP | Male | 30 | Right buccal mucosa |
| 3 | erosive OLP | Male | 51 | Left buccal mucosa |
| 4 | erosive OLP | Female | 52 | Left buccal mucosa |
| 5 | erosive OLP | Male | 64 | Right buccal mucosa |
| 6 | erosive OLP | Female | 53 | Right buccal mucosa |
| 7 | erosive OLP | Male | 54 | Right buccal mucosa |
| 8 | erosive OLP | Male | 48 | Left buccal mucosa |
| 9 | erosive OLP | Female | 48 | Right buccal mucosa |
| 10 | erosive OLP | Female | 49 | Right buccal mucosa |
| 11 | non-erosive OLP | Female | 23 | Ventrum of the tongue |
| 12 | non-erosive OLP | Male | 37 | Dorsum of the tongue |
| 13 | non-erosive OLP | Male | 47 | Right buccal mucosa |
| 14 | non-erosive OLP | Female | 46 | Ventrum of the tongue |
| 15 | non-erosive OLP | Female | 35 | Dorsum of the tongue |
| 16 | non-erosive OLP | Female | 31 | Right buccal mucosa |
