# Supplementary material for: 2‐Deoxy‐D‐glucose impedes T cell–induced apoptosis of keratinocytes in oral lichen planus
Source: J Cell Mol Med. 2021 Oct 21;25(21):10257–67. doi: 10.1111/jcmm.16964 (PMC8572795; doi:10.1111/jcmm.16964)
Supplement: Supplementary file 3 — Appendix S3 [file JCMM-25-10257-s004.docx]

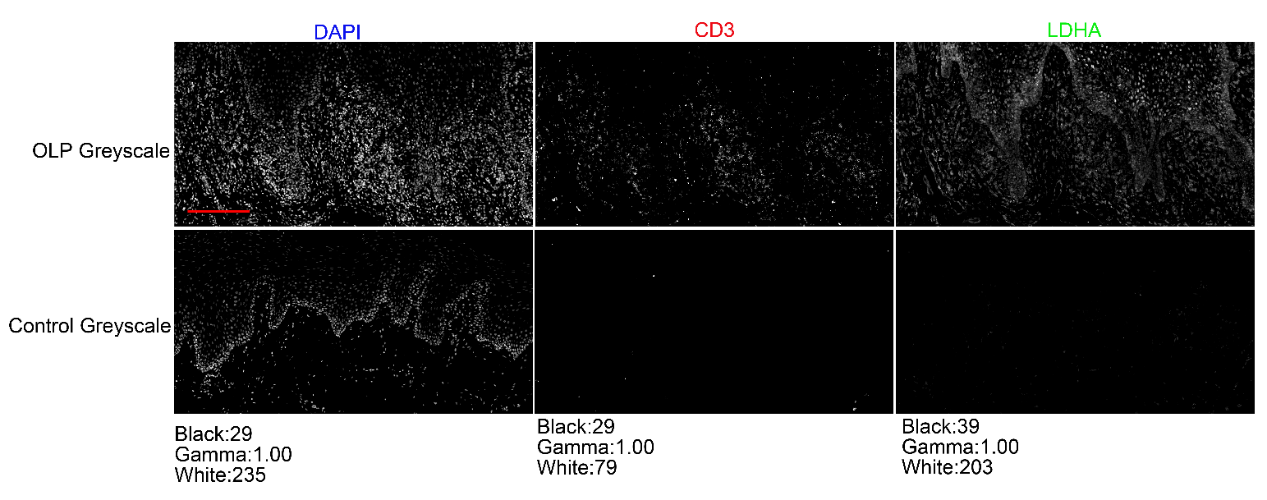


Appendix 3. The contrast parameters were matched between OLP group and Control group for Figure 1b. Magnification: ×200; Bar: 200 μm.
