# Supplementary material for: 2‐Deoxy‐D‐glucose impedes T cell–induced apoptosis of keratinocytes in oral lichen planus
Source: J Cell Mol Med. 2021 Oct 21;25(21):10257–67. doi: 10.1111/jcmm.16964 (PMC8572795; doi:10.1111/jcmm.16964)
Supplement: Supplementary file 4 — Appendix S4 [file JCMM-25-10257-s005.docx]

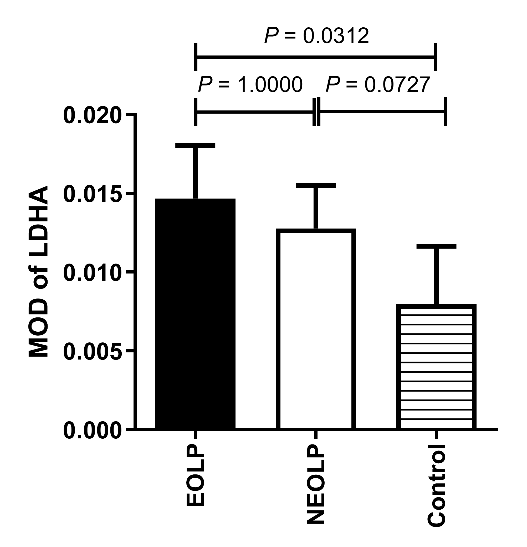


Appendix 4. OLP is clinically classified into reticular, plaque-like, papular, atrophy, erosive, and bullous forms. For comparative analysis of the differences between subtypes, OLP group were simplified as non-erosive and erosive forms. The expression of LDHA staining by immunohistochemistry in different subtypes of OLP lesions. EOLP, erosive OLP lesions, n = 6; NEOLP, non-erosive OLP lesions, n = 6; Control, normal oral mucosa tissues, n = 10.
