# Supplementary material for: 2‐Deoxy‐D‐glucose impedes T cell–induced apoptosis of keratinocytes in oral lichen planus
Source: J Cell Mol Med. 2021 Oct 21;25(21):10257–67. doi: 10.1111/jcmm.16964 (PMC8572795; doi:10.1111/jcmm.16964)
Supplement: Supplementary file 5 — Appendix S5 [file JCMM-25-10257-s003.docx]

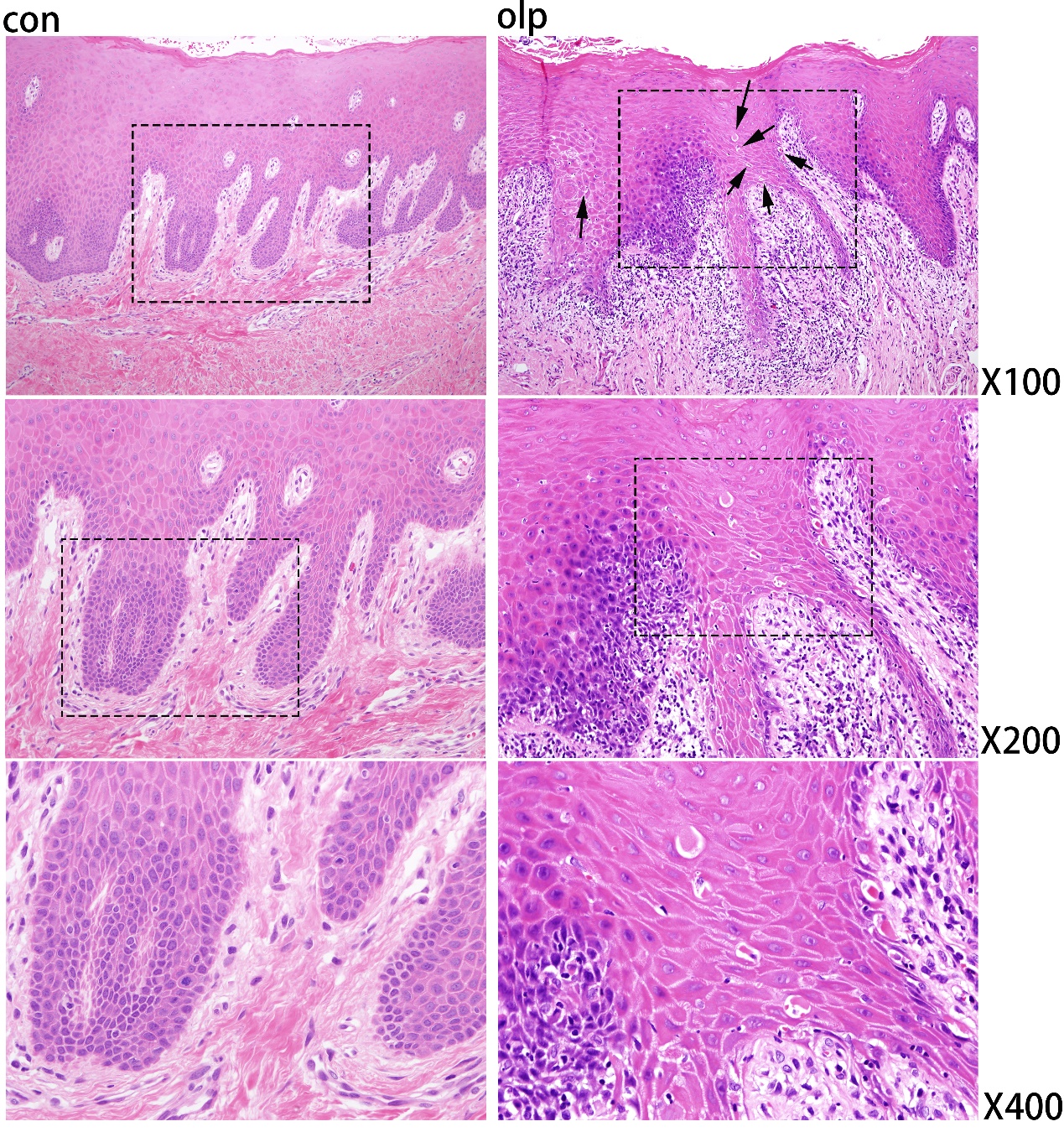


Appendix 5. OLP was featured with a band-like chiefly lymphocytic infiltrate in the lamina propria and basement membrane disruption. Colloid bodies (arrows) were seen in basement membrane of OLP tissues. Colloid bodies represented apoptotic keratinocytes.
