# Supplementary material for: 2‐Deoxy‐D‐glucose impedes T cell–induced apoptosis of keratinocytes in oral lichen planus
Source: J Cell Mol Med. 2021 Oct 21;25(21):10257–67. doi: 10.1111/jcmm.16964 (PMC8572795; doi:10.1111/jcmm.16964)
Supplement: Supplementary file 6 — Appendix S6 [file JCMM-25-10257-s002.docx]

Appendix 6. The early and late stages of apoptosis for OLP-derived T cells and keratinocytes in co-culture system. The differences were shown as “*” between DMSO (T cell) group, 2-DG (T cell) group, Rap (T cell) group and 2-DG + Rap (T cell) group, and as “#” between early apoptosis and late apoptosis. *, *P* < 0.05; **, *P* < 0.01; ***, *P* < 0.001; ###, *P* < 0.001.
